# Effects of Acute Endurance Exercise Performed in the Morning and Evening on Inflammatory Cytokine and Metabolic Hormone Responses

**DOI:** 10.1371/journal.pone.0137567

**Published:** 2015-09-09

**Authors:** Hyeon-Ki Kim, Masayuki Konishi, Masaki Takahashi, Hiroki Tabata, Naoya Endo, Shigeharu Numao, Sun-Kyoung Lee, Young-Hak Kim, Katsuhiko Suzuki, Shizuo Sakamoto

**Affiliations:** 1 Graduate School of Sport Sciences, Waseda University, Saitama, Japan; 2 Research Fellow of the Japan Society for the Promotion of Science, Tokyo, Japan; 3 Faculty of Sport Sciences, Waseda University, Saitama, Japan; 4 Faculty of Science and Engineering, Waseda University, Tokyo, Japan; 5 Japan Institute of Sports Sciences, Tokyo, Japan; 6 Department of Health and Sports Sciences, Kyoto Pharmaceutical University, Kyoto, Japan; 7 Waseda Institute for Sport Sciences, Waseda University, Tokyo, Japan; 8 Department of Sports and Physical Arts, Myongji University, Seoul, Korea; 9 Department of Oriental Martial Arts, Yongin University, Yongin, Korea; University of Lübeck, GERMANY

## Abstract

**Purpose:**

To compare the effects of endurance exercise performed in the morning and evening on inflammatory cytokine responses in young men.

**Methods:**

Fourteen healthy male participants aged 24.3 ± 0.8 years (mean ± standard error) performed endurance exercise in the morning (0900–1000 h) on one day and then in the evening (1700–1800 h) on another day with an interval of at least 1 week between each trial. In both the morning and evening trials, the participants walked for 60 minutes at approximately 60% of the maximal oxygen uptake ($\overset{\cdot }{\text{V}}{\text{O}}_{\text{2max}}$) on a treadmill. Blood samples were collected to determine hormones and inflammatory cytokines at pre-exercise, immediately post exercise, and 2 h post exercise.

**Results:**

Plasma interleukin (IL)-6 and adrenaline concentrations were significantly higher immediately after exercise in the evening trial than in the morning trial (*P* < 0.01, both). Serum free fatty acids concentrations were significantly higher in the evening trial than in the morning trial at 2 h after exercise (*P* < 0.05). Furthermore, a significant correlation was observed between the levels of IL-6 immediately post-exercise and free fatty acids 2 h post-exercise in the evening (r = 0.68, *P* < 0.01).

**Conclusions:**

These findings suggest that the effect of acute endurance exercise in the evening enhances the plasma IL-6 and adrenaline concentrations compared to that in the morning. In addition, IL-6 was involved in increasing free fatty acids, suggesting that the evening is more effective for exercise-induced lipolysis compared with the morning.

## Introduction

Hormonal changes, including increases in the plasma concentrations of several hormones (e.g., adrenaline and cortisol), occur in response to exercise and are known to have immunomodulatory effects [1,2]. In humans, several studies have reported that the secretion of interleukin (IL)-6 is possibly stimulated by catecholamines during exercise [3,4]. In addition, the effect of acute exercise on leukocytes is mediated by catecholamines [5,6]. The reduction of lymphocytes post exercise is also mediated by both catecholamines and cortisol [6]. These findings suggest that exercise stimulates immunoendocrine responses, with its effects on the inflammatory response in particular being mediated by activation of the sympathetic nervous system, and the hypothalamic-pituitary-adrenal axis [7].

The circadian system influences all aspects of physiological functions, including the endocrine, nervous, and immune systems [8–10]. Recently, inflammatory cytokines, including the tumor necrosis factor (TNF)-α, IL-6, and IL-1β, have been shown to exhibit diurnal variation in humans [11,12]. Moreover, increased plasma IL-6 has been reported to increase substrate metabolism [13]. In a previous study, an increase in the plasma concentration by an IL-6 infusion was shown to promote lipolysis and lipid oxidation [13]. In addition, catecholamines and cortisol concentrations are increased by acute endurance exercise, but the responses are different for morning and evening exercise [8,14–16]. Catecholamines are known to be involved in the secretory stimulation or inhibition of IL-6 [17–19]. Therefore, this response can be considered to have different possibilities depending on whether acute endurance exercise is performed in the morning or evening.

For the aforementioned reasons outlined, hormonal responses at different times of day may contribute to altering the inflammatory cytokine responses through acute endurance exercise. However, to the best of our knowledge, little is known about the effect of acute endurance exercise on inflammatory cytokine responses at a particular time of day. Moreover, a previous study showed that moderate-intensity exercise stimulates the immune system and may be somewhat responsible for exercise-related reduction in disease [20]. Therefore, further research is needed to investigate the role of acute endurance exercise on inflammatory cytokine responses at different times of day. The purpose of this study was to compare the effects of endurance exercise, performed either in the morning or evening, on inflammatory cytokine responses.

## Method

### Participants

Fourteen healthy males aged 21–30 years with no regular exercise training participated in this study after providing written informed consent. This study was conducted according to the guidelines laid down in the Declaration of Helsinki and was approved by the ethics committee of Waseda University (2011–906). Participants were recruited only if they met the following criteria: non-smoker, no known history of cardiovascular disease, body mass index (BMI) <30 kg/m^2^, and did not have any condition or take any medication known to affect inflammation.

### Preliminary measurements

On the subjects’ first visit to the laboratory, their height, weight, heart rate, and body fat percentage were measured by the same researcher. Participants initially performed an incremental exercise test to exhaustion on a treadmill (MAT-2700, Fukuda Denshi) to determine the maximal oxygen uptake ($\overset{\cdot }{\text{V}}{\text{O}}_{\text{2max}}$) as previously described [21]. During exercise, a 12-lead electrocardiogram was recorded (Stress Test System ML-6500; Fukuda Denshi), and heart rate was derived from the R-R interval. In addition, ratings of perceived exertion were determined at the end of each min of exercise. Breath-by-breath measurements were recorded throughout the exercise to monitor oxygen uptake ($\overset{\cdot }{\text{V}}{\text{O}}_{\text{2}}$) and carbon dioxide output ($\overset{\cdot }{\text{V}}{\text{CO}}_{\text{2}}$) by using an automated gas analysis system (AE-300S; Minato Medical Science, Osaka, Japan). The gas analyzers were calibrated with an O_2_ (15.3%), CO_2_ (5.1%) and N_2_ balance gas mixture, and the volume transducer was calibrated with a 2-L calibration syringe. Maximum effort was determined based on achieving a plateau of $\overset{\cdot }{\text{V}}{\text{O}}_{\text{2}}$ despite increasing workloads of meeting at least two of the following criteria: (1) a rating of perceived exertion >18, (2) attaining an age-predicted maximal heart rate (220 − age [in years]), and (3) achieving a high respiratory exchange ratio >1.15 [22]. Data from the maximal exercise test were used to determine 60% of $\overset{\cdot }{\text{V}}{\text{O}}_{\text{2max}}$ for each subject. Furthermore, the slope and speed of adjustment were calculated as the oxygen uptake of 60% $\overset{\cdot }{\text{V}}{\text{O}}_{\text{2max}}$ within 10 min from the start of exercise.

### Main trials

A randomized cross-over design was used. Each participant underwent two laboratory-based trials in random order: (1) morning trial (0900–1000 h), and (2) evening trial (1700–1800 h). The interval between trials was at least 1 wk. Each participant walked at 60% $\overset{\cdot }{\text{V}}{\text{O}}_{\text{2max}}$ on a treadmill for 60 min. On the day of the trials, all participants ate the same diet 3 h before exercise. The meal was 2,430 kJ, and energy was derived from 34.4% fat, 59.9% carbohydrates, and 5.7% protein. All subjects were instructed to maintain their usual daily dietary, sleep, and physical activity patterns during the entire study, and they repeated their dietary intake, sleep, and physical activity patterns during the day before each trial. Subjects were also instructed to abstain from transitory strenuous physical exercise and alcohol intake for at least 2 days before each trial.

### Blood collection and analysis

Venous blood samples were collected from each participant before and immediately after exercise, as well as at 2 h after exercise. Blood samples were collected in a 6-mL tube containing thrombin and a heparin-neutralizing agent, a 7-mL tube containing ethylenediaminetetraacetic acid (EDTA)-Na_2_, and a 2-mL tube containing EDTA-Na_2_. Hemoglobin and hematocrit were determined on EDTA-treated venous blood using an automatic blood cell counter (pocH-100i, Sysmex). Remaining tubes were centrifuged at 3,500 rpm at 10 min after collection, and plasma and serum were stored at -80°C until the assay. Plasma TNF-α, IL-6, and IL-1β were measured using commercial solid-phase, enzyme-linked immunosorbent assay (High sensitivity, Quantikine, R&D System, Minneapolis, MN). Plasma adrenaline and noradrenaline concentrations were measured using high-performance liquid chromatography. Plasma concentrations of cortisol were measured by radioimmunoassay. Serum C-reactive protein (CRP) concentrations were measured by latex turbidimetric immunoassay. Serum free fatty acids concentrations were analyzed using an enzymatic colorimetric method. Plasma adrenaline, noradrenaline, cortisol, and serum CRP were analyzed by SRL, Inc. (Tokyo, Japan). Changes in plasma volume during the acute bout of exercise were calculated using the method outlined by Dill and Costill, and were used for the collection of blood markers [23].

### Statistical analysis

All data were presented as a mean ± standard error. All blood parameters were tested for normal distribution using the Kolmogorov-Smirnov test. The distribution of these parameters did not differ significantly from normal. To compare changes in plasma hormones, plasma IL-6, TNF-α, IL-1β, and serum CRP concentrations between trials over time, two-way repeated measures analysis of variance was used with the trial and time as factors. When significant main or interaction effects were detected, we used the Bonferroni Method for post-hoc comparisons. Pearson’s product-moment correlation coefficient was calculated to determine the relationship between IL-6 and free fatty acid. All statistical significance was set at *P* < 0.05. Data analysis was performed using PASW Statistics 18 software (SPSS Japan Inc.).

## Results

### Participants’ characteristics

The participants’ characteristics are shown in Table 1.

**Table 1:** Baseline physical characteristics (N = 14).

	Mean ± SE
Age	24.3 ± 0.8
Height (cm)	174.8 ± 1.7
Body mass (kg)	71.6 ± 2.7
BMI (kg/m^2^)	23.4 ± 0.7
%fat	17.5 ± 1.3
$\overset{\cdot }{\text{V}}{\text{O}}_{\text{2max}}$	48.7 ± 2.2

Value are mean ± SE, BMI, body mass index.

### Plasma hormone responses

**Fig 1:**
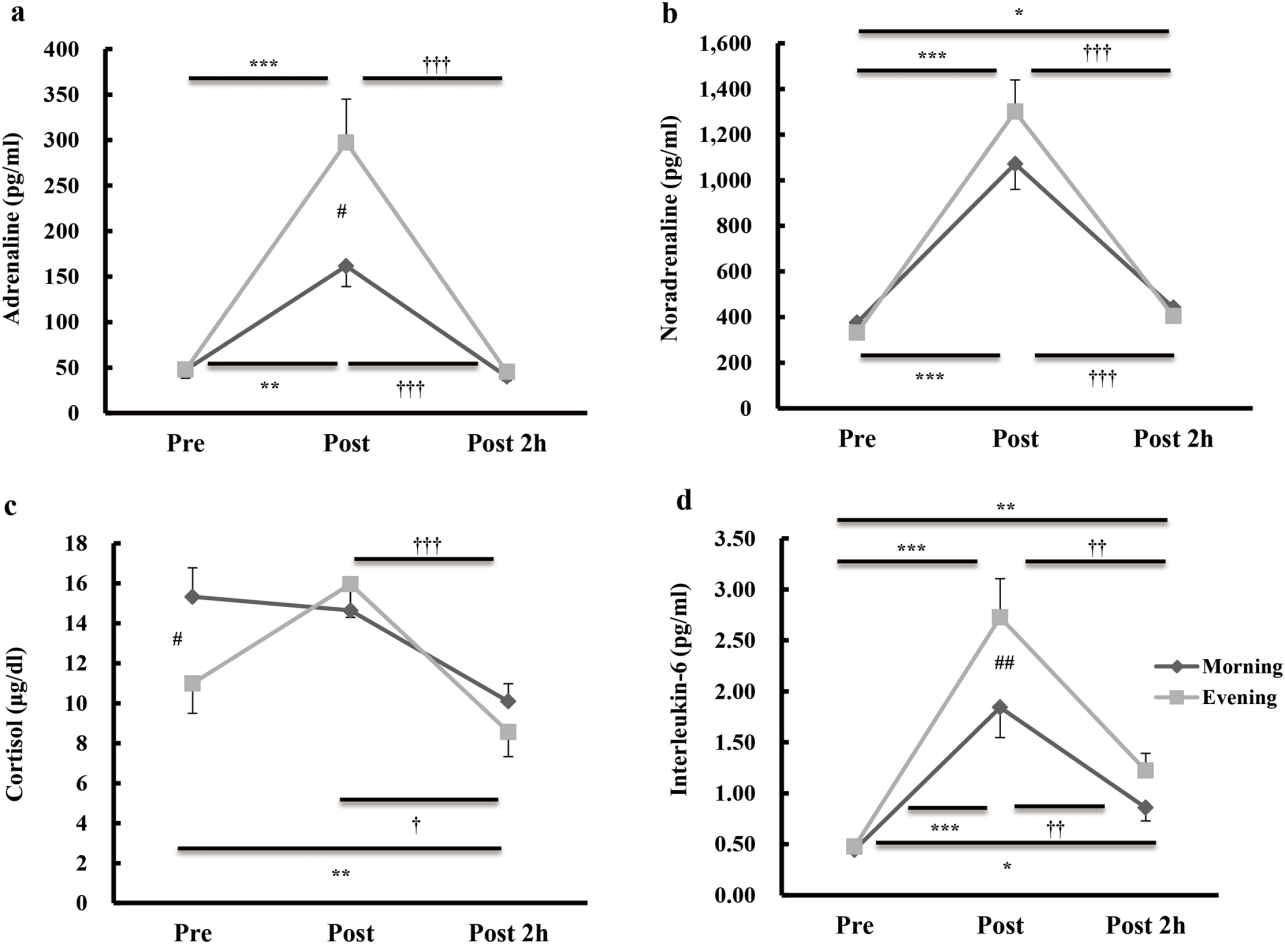
Changes in plasma hormone and IL-6 during morning and evening of acute endurance exercise. Plasma concentrations of adrenaline (a), noradrenaline (b), cortisol (c) and interleukin-6 (d) before (Pre), immediately after (Post), and 2 h after (Post 2 h) exercise. Data represent the mean ± standard error. * *P* < 0.05, ** *P* < 0.01, and *** *P* < 0.001, compared with values at pre-exercise. ^†^ *P* < 0.05, ^††^ *P* < 0.01, and ^†††^ *P* < 0.001, compared with values immediately after exercise. ^#^ *P* < 0.05 and ^##^ *P* < 0.01, significant difference between the morning and evening values.

Significant trial × time interactions were found for changes in plasma adrenaline, noradrenaline, and cortisol concentrations (*P* < 0.01, *P* < 0.05, and *P* < 0.05, respectively). Plasma adrenaline concentrations were significantly higher immediately after exercise in the evening trial than in the morning trial (*P* < 0.01). However, plasma noradrenaline concentrations were not significantly different between the trials. The plasma cortisol concentration was significantly higher before exercise in the morning trial than in the evening trial (*P* < 0.05) (Fig 1).

### Plasma inflammatory cytokines and serum CRP

Significant trial × time interactions were found for changes in plasma IL-6 and TNF-α (*P* < 0.05, both). Plasma IL-6 concentrations were significantly higher immediately after exercise in the evening trial than in the morning trial (*P* < 0.01) (Fig 1d). Conversely, plasma TNF-α concentrations were significantly higher pre-exercise in the morning trial than in the evening trial (*P* < 0.05, both). There was no significant trial × time interaction for changes in the plasma IL-1β and serum CRP concentrations (Table 2).

**Table 2:** Change in plasma cytokines and serum CRP in the morning and evening (N = 14).

	Morning			Evening		
	Pre	Post	Post 2h	Pre	Post	Post 2h
TNF-α (pg/ml)	0.59 ± 0.14 ^#^	0.56 ± 0.13	0.60 ± 0.12	0.52 ± 0.12	0.60 ± 0.12	0.62 ± 0.14
IL-1β (pg/ml)	0.18 ± 0.05	0.14 ± 0.03	0.14 ± 0.02	0.14 ± 0.02	0.19 ± 0.04	0.16 ± 0.03
CRP (mg/dl)	0.04 ± 0.01	0.04 ± 0.01	0.04 ± 0.01	0.05 ± 0.01	0.05 ± 0.01	0.05 ± 0.01

Value are mean ± SE, TNF-α, Tumor necrosis factor-alpha; IL-1β, Interleukin-1β; CRP, C-reactive protein.

^#^ *P* < 0.05 compared with level (Pre) in the Evening trial.

### Lipid metabolism

**Fig 2:**
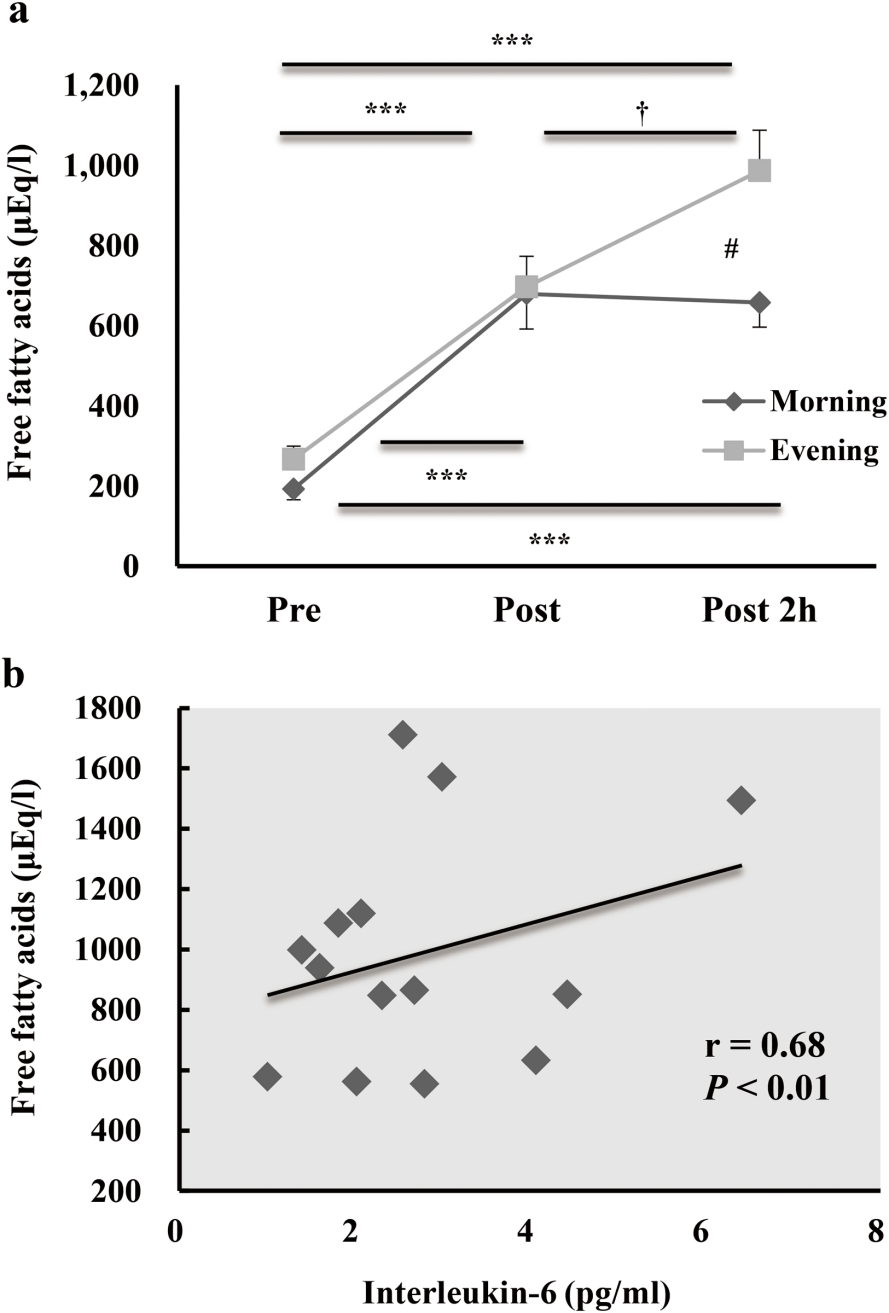
The relationship between the free fatty acids and IL-6 during morning and evening of acute endurance exercise. Serum concentrations of free fatty acids (a) before (Pre), immediately after (Post), and 2 h after (Post 2 h) exercise. Data represent the mean ± standard error. *** *P* < 0.001, compared with values at pre-exercise. ^†^ *P* < 0.05, compared with values immediately after exercise. ^#^ *P* < 0.05, significant difference between the morning and evening values. The relationship between levels of interleukin-6 immediately post exercise and free fatty acids 2 h post exercise in the evening (r = 0.68, *P* < 0.01) (b).

Serum free fatty acids concentrations were significantly higher in the evening trial than in the morning trial at 2 h after exercise (*P* < 0.05). Furthermore, a significant correlation was observed between levels of IL-6 immediately post exercise and free fatty acids 2 h post exercise in the evening (r = 0.68; *P* < 0.01) (Fig 2).

## Discussion

This study is one of the first to examine the effects of acute endurance exercise on inflammatory cytokine responses at different times of day. The unique aspects of our study are as follows. First, in 60-min periods of acute endurance exercise in the morning and evening, the concentrations of plasma IL-6 were significantly higher in the evening trial than in the morning trial immediately after exercise. Second, plasma adrenaline concentrations were significantly higher immediately after exercise in the evening trials than in the morning trials. Third, free fatty acids after exercise, which were shown to be associated with IL-6, were more enhanced in the evening trials. These findings show that inflammatory cytokine and hormone responses to acute endurance exercise are more enhanced in the evening than in the morning, and this has been suggested to influence the difference between morning and evening lipolytic responses.

One of the interesting findings in this study was that a 60-min period of acute endurance exercise induced a significantly higher increase in concentrations of plasma adrenaline and IL-6 in the evening trials in comparison with the morning trials. Adrenaline increases the concentration of IL-6 [17,18]. However, noradrenaline affects IL-6 release in both inhibition and activation [19]. In addition, catecholamine responses to exercise differ in the morning and evening, with it being reported that they are higher in the evening than in the morning [8,14]. Plasma adrenaline concentrations in this study were significantly higher immediately after exercise in the evening than in the morning, which matches previous studies [8,14]. However, plasma noradrenaline concentrations were not significantly different between the trials. In this study, differences in adrenaline concentrations could have been seen due to differences in blood flow in the morning and evening during the trials of acute endurance exercise [24]. However, an explanation for the different adrenaline responses to acute endurance exercise in the morning and evening remains elusive. IL-6 is a multifunctional cytokine that plays an important role in immune regulation and inflammation, depending on factors such as exercise intensity and duration, which are increased during and post exercise [25]. IL-6 is secreted from adipose tissue and muscle cells by the contraction of skeletal muscles during exercise, and it contributes to substrate metabolism through the effect of autocrine and paracrine action [26,27]. In previous studies, an increase in IL-6 has been reported to enhance lipolysis and increase free fatty acids concentrations [28,29]. Furthermore, the enhancement of lipolysis has been shown to be maintained up to 2 h after injection of IL-6 [28,29]. In this study, free fatty acids were observed to have significantly higher concentrations in the evening compared to the morning after a period of exercise for 2 h. Additionally, a significant correlation was observed between levels of IL-6 immediately post exercise and free fatty acids 2 h post exercise in the evening. However, no significant correlation was observed with the immediate post exercise period of the morning trial. Therefore, contributions to the enhanced lipolysis of the increased plasma IL-6 due to acute endurance exercise is considered to be higher in the evening compared to the morning. In addition, increases in the plasma concentrations of IL-6 due to acute endurance exercise is not dependent on the inflammatory cascade of TNF-α, and it has been shown to be produced by skeletal muscle contraction [30]. In fact, there were no significant changes in the TNF-α response during the morning and evening trials of acute endurance exercise in the present study. Thus, it can be presumed that the IL-6 response to acute endurance exercise is partly attributed to skeletal muscle contraction. From these findings, the concentrations of plasma adrenaline and IL-6 to acute endurance exercise in the morning and evening were significantly higher in the evening than in the morning, which suggests that adrenaline may be involved in the changes of IL-6. Moreover, plasma IL-6 increases resulting from acute endurance exercise have been suggested to influence increased lipolysis.

It is meaningful that the elevated plasma IL-6 observed in the present study enhanced lipolysis after exercise. Plasma IL-6 increased immediately after exercise in the present study by an extent equivalent to observations in previous research [26]. IL-6 has a lipid metabolism-enhancing action [26,27]. However, the injection of recombinant human IL-6 has often been used in research [13,31], and it is not clear how lipid metabolism is affected by fluctuations in IL-6 caused by exercise. With exercise in the present study, plasma IL-6 increased modestly, suggesting that lipolysis after exercise is enhanced.

Differences were not seen in the response of plasma cortisol during the morning and evening trials of acute endurance exercise. Cortisol has been shown to be a major factor in the neuroendocrine regulation of the immune system, having an anti-inflammatory effect, and it has been reported to be negatively correlated with the production of a number of inflammatory cytokines [32]. In addition, the response of cortisol to exercise has been reported to be higher in the morning in comparison with the evening [16]. However, there were no significant differences in the responses of cortisol between the trials in the current study. The response of cortisol to exercise has been shown to be effected by factors such as exercise intensity, duration, and diet [33,34]. In previous studies, it was considered that food, which had not been controlled, affected the cortisol response. However, the present study controlled food as well as the exercise duration and intensity pre-exercise. Therefore, our study findings suggest that the response of cortisol to acute endurance exercise may not be affected by the time of day such as the morning and evening.

There are several limitations to this study. First, subjects in our study were healthy young men. Our findings do not apply to other individuals such as obese men and women, or populations with low-grade inflammation. Second, it is impossible to rule out that the hormone and cytokine response during morning and evening exercise was impacted by the circadian rhythm, thus it would be necessary to set conditions without morning and evening exercise and more stringently investigate the impact with respect to morning and evening exercise. However, regarding the hormone and cytokine response in the present study, the response from morning and evening exercise was greater than the magnitude of variation that is observed due to the circadian rhythm, and therefore the conclusions of the present study are believed to be valid. Finally, the pre-experiment diet was regulated throughout the present study so there was no precise assessment of the sleep time on the day before the experiment. A lack of sleep can potentially impact metabolism or the immune response so further investigation needs to be performed along with regulating sleep.

## Conclusions

In conclusion, our study findings demonstrated that acute endurance exercise in the evening enhanced more plasma IL-6 and adrenaline concentrations in comparison with that in the morning. Plasma IL-6 also functions as a hormone that acts on the enhancement of lipolysis, and it has been indicated to increase the blood levels of free fatty acids 2 h after exercise. From these findings, the effects of exercise in the evening on enhanced lipolysis seem to be more beneficial than exercise in the morning.
